# Fabric topological haptic proxy for interactive virtual reality

**DOI:** 10.1093/nsr/nwag041

**Published:** 2026-01-23

**Authors:** Zhiyang Hu, Tianzhan Liang, Yuchen Wu, Haoyu Wang, Minyu Zhou, Xinyan Lin, Leheng Chen, Shuqun An, Haojie Zhao, Yongqi Lou, Guoqing Zhang, Hongguo Gao, Fujie Li, Yuwen Zhu, Ling Zhang, Guanglin Zhang, Liang-Wen Feng, Qi Wang, Hengda Sun, Xinge Yu, Hongzhi Wang, Jun Chen, Xiang-Chen Li, Gang Wang

**Affiliations:** State Key Laboratory of Advanced Fiber Materials, College of Materials Science and Engineering, Donghua University, Shanghai 201620, China; College of Design and Innovation, Tongji University, Shanghai 200092, China; College of Information Sciences and Technology, Donghua University, Shanghai 201620, China; State Key Laboratory of Advanced Fiber Materials, College of Materials Science and Engineering, Donghua University, Shanghai 201620, China; State Key Laboratory of Advanced Fiber Materials, College of Materials Science and Engineering, Donghua University, Shanghai 201620, China; State Key Laboratory of Advanced Fiber Materials, College of Materials Science and Engineering, Donghua University, Shanghai 201620, China; College of Design and Innovation, Tongji University, Shanghai 200092, China; State Key Laboratory of Advanced Fiber Materials, College of Materials Science and Engineering, Donghua University, Shanghai 201620, China; State Key Laboratory of Advanced Fiber Materials, College of Materials Science and Engineering, Donghua University, Shanghai 201620, China; College of Design and Innovation, Tongji University, Shanghai 200092, China; Yuyue Home Textile Co., Ltd, Binzhou 256623, China; Yuyue Home Textile Co., Ltd, Binzhou 256623, China; Shandong Xinyue Health Science and Technology Co., Ltd, Binzhou 256600, China; State Key Laboratory of Advanced Fiber Materials, College of Materials Science and Engineering, Donghua University, Shanghai 201620, China; College of Polymer Science and Engineering, Sichuan University, Chengdu 610065, China; College of Information Sciences and Technology, Donghua University, Shanghai 201620, China; Key Laboratory of Green Chemistry & Technology, Ministry of Education, College of Chemistry, Sichuan University, Chengdu 610064, China; College of Design and Innovation, Tongji University, Shanghai 200092, China; State Key Laboratory of Advanced Fiber Materials, College of Materials Science and Engineering, Donghua University, Shanghai 201620, China; Henan Academy of Sciences, Zhengzhou 450046, China; Department of Biomedical Engineering, City University of Hong Kong, Hong Kong 999077, China; State Key Laboratory of Advanced Fiber Materials, College of Materials Science and Engineering, Donghua University, Shanghai 201620, China; Department of Bioengineering, University of California, Los Angeles, Los Angeles, CA 90095, USA; China Institute of Sport Science, Beijing 100061, China; State Key Laboratory of Advanced Fiber Materials, College of Materials Science and Engineering, Donghua University, Shanghai 201620, China; Yuyue Home Textile Co., Ltd, Binzhou 256623, China; China Institute of Sport Science, Beijing 100061, China

**Keywords:** haptic proxies, topological fabrics, smart textiles, virtual reality

## Abstract

Physical objects serving as haptic proxies offer a promising approach to enrich tactile experience in virtual reality. However, conventional haptic proxies are hampered by prohibitive costs, limited reusability, and the logistical burden of creating and storing numerous object-specific models. Here, we introduce a fabric-based topological haptic proxy (FTHP) that functions as a programmable and universal interface. Our approach synergistically integrates origami-inspired topological constraints with triboelectric sensor yarns. The engineered topological design, featuring heterogeneous rigid and flexible segments, restricts deformation pathways, ensuring structural stability and generating distinct, classifiable electrical signals for different interactions. This allows a single, reusable FTHP to be dynamically reconfigured into multiple functional states (e.g. a flat touchpad or various 3D geometric controllers), bypassing the need for a rigid one-to-one correspondence between physical props and pre-stored virtual assets. Integrated with a convolutional neural network (CNN), the system achieves a 92.4% recognition accuracy across 14 distinct actions and 3 interaction modes. The FTHP presents a scalable and versatile platform for high-fidelity haptic interaction, advancing the design of more immersive and accessible virtual reality (VR) systems.

## Teaser

A reconfigurable fabric-based topological haptic proxy, integrating origami-inspired rigid–flex structures with triboelectric yarn sensing and CNN recognition, enables a single textile interface to deliver versatile, high-fidelity VR interactions without object-specific props.

## INTRODUCTION

Haptic feedback is pivotal for enhancing user immersion, interaction fidelity and spatial cognition in virtual reality (VR) systems by enabling tactile perception and manipulation of virtual objects. Recent advances in haptic interfaces have expanded application scenarios and multifunctionality [1,2], including wearable devices [3], graspable systems [4] and haptic displays [5]. Nevertheless, delivering high-fidelity tactile sensations remains challenging, particularly in accurately discerning object properties (e.g. shape, structure, boundaries) through touch [6], which are essential for tangible interaction and realistic manipulation. This limitation underscores an urgent need for advanced haptic technologies to bridge the realism gap in VR. Passive haptic proxies, which leverage physical objects to simulate tactile feedback, offer a promising solution [7–9], yet the core challenge of conventional passive haptics is the principle of one-to-one correspondence, where each virtual object requires a physically registered, often custom-made, 3D replica [10,11]. This imposes prohibitive costs, limited reusability and scalability constraints in model production/storage [12,13]. Consequently, these constraints have largely confined passive haptics to niche applications, hindering their widespread adoption. Exploring universal alternatives represents a promising approach. Ideally, a single reconfigurable prop could emulate multiple physical models through controlled deformation. Prior work has demonstrated that foldable rigid columns with linkages can approximate simple geometric objects by exploiting visual dominance in VR [14]. However, rigid structures constrain deformation pathways, whereas overly flexible ones introduce signal ambiguity, motivating the search for a design that balances structural stability with reconfigurability.

Textile-based sensors have emerged as a compelling alternative, offering soft integration, multifunctionality and adaptability to various shapes and textures [15–17]. While prior textile research has largely focused on active haptic feedback via electrical stimulation [18–21], developing textiles as passive, shape-adaptive proxies remains largely underexplored. Programmable textiles, with their inherent deformability and portability, offer a promising platform for passive haptic proxies [22,23], yet the very compliance that makes textiles advantageous is also the source of two critical challenges. On a physical level, their inherent softness impedes stable shape retention during interaction, compromising feedback reliability. The physical instability translates directly into an informational one; unconstrained deformation pathways generate a noisy and ambiguous array of electrical signals for a single target shape, necessitating object-specific algorithms that contradict the goal of a model-free system [24–26]. However, the weave structure of textiles allows for configuration of rigid regions through simple sewing or embroidery, thereby balancing the rigidity and flexibility. This advantage leads us to consider the potential of textiles as high-performance passive haptic proxies [27].

Here, we introduce a fabric-based topological haptic proxy (FTHP) as a robust solution to these challenges. Inspired by origami [28–30], we architect the FTHP with heterogeneous rigid segments [e.g. polytetrafluoroethylene (PTFE), nylon] interconnected by flexible fabric ‘hinges’. The engineered topology constrains the fabric’s vast deformation space into a predictable set of pathways, which is the key to simultaneously guaranteeing shape stability while encoding complex physical interactions into simple, classifiable electrical signatures. The reliable detection of these signatures is enabled by a high-performance triboelectric yarn, created by engineering a triboelectrically active polyvinylidene fluoride (PVDF) sheath and a conductive silver-plated nylon core into a dual-layer S/Z-twist architecture. This design yields a significant output enhancement over single-layer counterparts (95.6%), coupled with an ultrarapid response and excellent operational durability. This synergistic integration of concept, structure and material culminates in a complete, highly robust haptic proxy system. By leveraging a lightweight convolutional neural network (CNN), our system translates the yarn signals into high-level commands (92.4% accuracy across 14 actions and 3 modes), enabling a single FTHP to fluidly transition between roles—from a flat touchpad to a 3D geometric controller—without physical–virtual model synchronization. Ultimately, the FTHP framework redefines the haptic proxy from a static physical mimic to a dynamic, information-rich interface, paving the way for more scalable, accessible and truly immersive virtual experiences.

## RESULTS AND DISCUSSION

### Design of the FTHP

**Figure 1.**
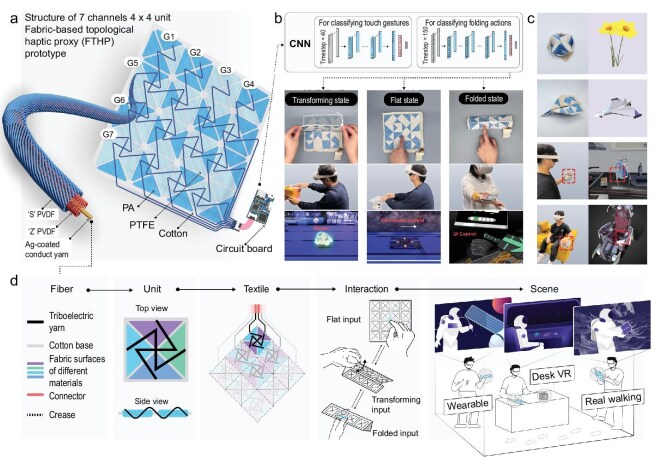
Overview of FTHP design. (a) Schematic diagram of the FTHP structure with seven channels and a 4 × 4-unit layout. Each triboelectric yarn consists of an ‘S/Z’ twisted PVDF core-sheathed silver-plated nylon yarn. Seven sensing channels are embedded in topological unit bases and connected to a PCB. (b) User interactions are classified into multiple specific actions within three predefined interaction modes—transforming state, flat state, and folded state—through a customized algorithm. (c) FTHP images of forming various geometric shapes and interacting within everyday environments. (d) The interrelation between different levels of the FTHP system. By extending and piecing together these topological units, a topological fabric is created that allows users to interact with VR through three types of inputs: flat input, transforming input, and folded input. The FTHP systems are expected to be utilized in wearable, desktop and actual walking VR interaction scenarios for daily VR usage.

The FTHP consists of three main components, as depicted in Fig. 1a. Seven channels for triboelectric signal recording, 16 topological units each containing heterogenous rigid segments for deformation and supporting, and a printed circuit board (PCB) for signal processing and transmission. Each channel generates electrical signals responsive to mechanical interactions (e.g. touch, deformation). A CNN framework decodes and classifies motion-signals gesture recognition [31–33], mapping user interactions to predefined commands across three FTHP states (transforming, flat, and folded) (Fig. 1b).

The topological units employ cotton fabric as deformable ‘hinges’ between rigid PTFE or nylon segments (Fig. S1). This heterogeneous material configuration generates unit-specific triboelectric signatures. Sensing channels comprise core–sheath triboelectric yarns—PVDF-coated silver-plated nylons with dual S/Z-twist [34,35], to capture dynamic interactions at minimal power consumption. These yarns were embroidered onto topological units via nested iteration space-filling patterns, ensuring full signal coverage during deformation. This architecture allows the FTHP to flexibly attach to objects of varying size, enabling real-world-to-VR transformation. (Fig. 1c and d).

### Construction of topological units

**Figure 2.**
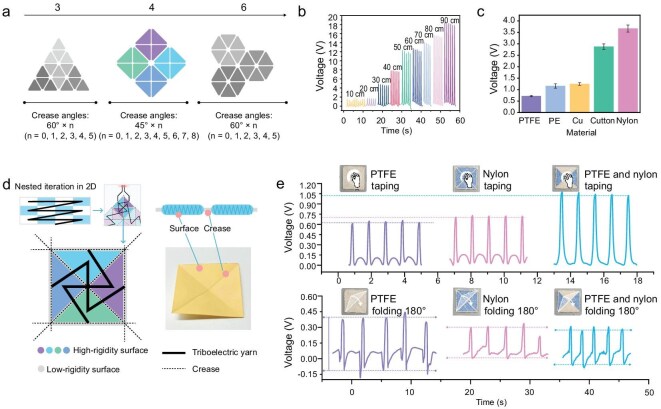
Characteristics of the topological unit. (a) The number and angles of creases in various polygonal layouts of tiling. (b) The strengths of triboelectric yarn signals at different contact lengths ranges from 10 to 90 cm. (c) The impact of different materials on topological unit to triboelectric signal strength. Each error bar is based on five data points. The error bars for each dataset are derived from five samples prepared under the same conditions. (d) Schematic of the design of the topological unit and triboelectric yarn. (e) Signal differences produced by topological units with different rigid surface materials. Topological units made of three materials generate voltage signals of 0.61, 0.71 and 1.03 V when slapped by a hand, and 0.46, 0.31 and 0.33 V when folded 180°. Each dataset uses the same 30 cm of triboelectric fiber.

The inherent compliance of fabrics permits diverse deformation pathways [36,37], complicating shape identification as various paths yield non-unique electrical signals per target geometry. Drawing from origami and tessellation principles, a cubic lattice structure was engineered for topological units via embroidery. Each square unit divides into four rigid isosceles right-angle triangles, isolated by foldable cotton ‘hinges’ (Fig. S2). This design follows the box-pleat tiling principle, where an *n* × *n* 2D grid layout allows for the assembly of *O*(*n*) 3D topological units (e.g. unit cubes). This scaling law illustrates the relationship between the planar fabric dimensions and the achievable 3D structural complexity. (Fig. 2a) [28]. Similar to origami transformations, the resulting 2D or 3D geometric structures after folding can simulate spatial geometries [38,39] and the folding actions during transformation are able to reflect interactive behaviors [40].

Triboelectric yarns traversing each unit must precisely record deformation-induced signals, demanding stringent mechanical/electrical properties to prevent signal loss from impedance mismatch or structural defects. We implemented a conjugated-twist strategy fabricating PVDF yarns with S/Z-bilayer twisting; after electrospinning PVDF onto silver-plated nylon, a second PVDF layer was applied in the opposite twist direction (Fig. S3). The selection of this specific S/Z architecture over unidirectional twisting (e.g. S/S twist) can yield both mechanical and electrical benefits. Mechanically, unidirectional twisting tends to induce residual torque and loose wrapping, leading to eccentric cross-sections and sheath stripping during the high-speed embroidery process. Conversely, the reverse-direction Z-twist exerts a compressive force on the inner S-twist layer, creating a self-tightening structure. This eliminates internal gaps and ensures a uniform, concentric diameter, which is essential for surviving the mechanical stresses of machine sewing. Electrically, the triboelectric yarn operates via triboelectric charging and electrostatic charge transfer modes (Fig. S5). The voltage output characteristics of triboelectric nanogenerators (TENGs) are given by [41,42]:


(1) \begin{eqnarray*}
V = - \frac{1}{{{C}_{(x)}}}Q + {V}_{oc}(x),
\end{eqnarray*}

where


(2) \begin{eqnarray*}
C(x) = \frac{{{\varepsilon }_0S}}{{{d}_0 + x(t)}},
\end{eqnarray*}

and


(3) \begin{eqnarray*}
{V}_{oc}(x) = \sigma x(t)/{\varepsilon }_0.
\end{eqnarray*}

Here, *V* is the terminal voltage across the electrodes, *Q* is the transferred charge, *C(x)* is the separation-dependent capacitance, *V_oc_*(*x*) is the open-circuit voltage, $\varepsilon $_0_ is the vacuum permittivity, *S* is the effective contact area, *d*_0_ represents the equivalent thickness of the dielectric layer, *x*(*t*) is the time-varying separation distance between the triboelectric surfaces (*t* denotes time), and *σ* is the triboelectric surface charge density.

Crucially, the 2L architecture introduces a dual-layer geometry that effectively doubles the structural dimension compared to the 1L counterpart. This geometric amplification naturally translates to a larger effective separation distance, *x*(*t*). Meanwhile, the S/Z dual-twist architecture of the triboelectric yarn provides significant structural stability, minimizing deformation under pressure and maintaining a relatively constant effective contact area (*S*). Furthermore, the dimensional variations of the dielectric layer in our system occur at the micro-to-millimeter scale, falling within the regime where classical electrostatic linearity dominates. Since Equation (3) dictates that open-circuit voltage is directly proportional to *x*(*t*), this increased separation distance theoretically accounts for the electrical enhancement, aligning with the ∼95.6% higher voltage output observed in the 2L yarn (Fig. S6).

Prior studies indicates that triboelectric nanogenerator (TENG) voltage magnitude positively correlates with frictional contact area [43,44]. For the 2L-triboelectric yarn, contact area changes during deformation are reflected in voltage signals. Measurements across contact length (10–90 cm) demonstrate a linear correlation between length and voltage output, with signals increasing from ∼2 to ∼18 V (Fig. 2b). Additional tests characterize voltage, current and power of a 30-cm yarn under varying loads, showing a peak power output of 1.50 μW at 500 MΩ, indicating an internal impedance of ∼16.7 MΩ cm^−1^ (Fig. S7). Abrasion and water-resistance tests confirm consistent electrical performance over prolonged use (Fig. S8). Under standard conditions, the yarn maintains stable output through 27 000 deformation cycles with a 100 ms response time (Figs S9 and S10), establishing reliable long-term recognition capabilities for topological units.

To ensure distinct electrical outputs, a matching substrate for the triboelectric yarn is essential. As shown in Fig. 2c, PTFE paired with nylon generates significant signal differentials, enabling material-driven variability to decouple signatures across the 16 units. Accordingly, we constructed four ridge segments with different PTFE/nylon configurations, interconnected by 2L-triboelectric yarns to form each topological unit, as shown in Fig. 2d. The substrate-dependent triboelectric mapping allows distinct unit responses to identical stimuli. When tapped, the pure PTFE unit shows an output of 0.61 V; the output voltages for the nylon unit and alternating unit are 0.71 and 1.03 V, respectively. During 180° folding, the pure PTFE generates 0.46 V, while the nylon and alternating units exhibit 0.31 and 0.33 V (Fig. 2e). Thus, the origami-structured units with differentiated electrical signatures provide reliable state-specific response.

### Integration of topological units into FTHPs

**Figure 3.**
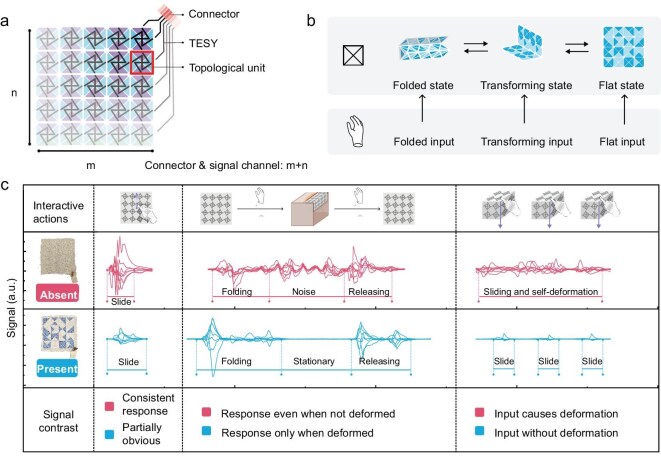
FTHP design. (a) Schematic illustration of expanding topological units into a rectangular FTHP. TESY, triboelectric sensing yarn (i.e., the PVDF-coated, silver-plated nylon coresheath triboelectric yarn used as the sensing/connection channel). (b) Schematic of FTHP’s operational states. (c) Signal comparison under the same interaction for a 4 × 4 scale system with either absent or present topological units. The FTHP system demonstrates more stable and clearer signal outputs in response to flat input, folded input and transforming input, compared to those of FHP.

The FTHP substrate integrates 16 topological units. Triboelectric yarns from each unit merge into shared sensing channels when interconnected at entry/exit points. For a rectangular FTHP of size m × n, this configuration requires m × n topological units and maximally m + n triboelectric yarns (Fig. 3a).

Connection configurations of inter-unit yarns critically influence signal detection fidelity, particularly in mapping physical interactions. Inspired by nested space-filling iteration, we designed an asymmetric connection layout that avoids signal uniformity caused by symmetry (Fig. S11), enabling differentiation of interactions at symmetrical positions. Conventional configurations failed to distinguish planar gesture pairs (e.g. ‘upward-leftward’ vs. ‘rightward-downward’), likely due to symmetric sensor channel distribution in planar expansions. This underscores a key FTHP design principle that sensor channel arrangements should eliminate shared symmetry axes to prevent ambiguous recognition of symmetric deformations.

FTHP classifies captured interactions into three primary states based on deformable interface: flat state, folded state and transforming state. Flat input is for interactions with the unfolded FTHP, folded input is for engagement with 3D-form FTHP, and transforming input is for transition between the ‘flat’ and ‘folded’ states (Fig. 3b). To validate that topological units combined with triboelectric yarn enhance recognition accuracy across all states, we fabricated a 4 × 4 topological fabric with seven sensing channels (Fig. S12). A fabric-based haptic proxy (FHP) not containing topological units was concurrently prepared. Compared with the FTHP, the FHP contains a sheet of pure cotton with the same connected way of triboelectric yarns.

Analysis of typical actions under three states reveals a distinct response, demonstrating FTHP’s superior functionality (Fig. 3c). This qualitative improvement is further corroborated by quantitative signal-to-noise ratio (SNR) and peak-amplitude analyses (Fig. S13), which show suppressed baseline fluctuations and higher cross-channel SNR when topological units are present. Due to the rigid topological structure, FTHP in the flat state exhibits a response concentrated in the area where the finger touches, without undergoing any physical deformation. When the FTHP is applied in the transforming state, this structure results in a limited and predictable deformation path, allowing the deformation signals to be accurately recognized and sorted. In the folded state, the structure provides support to maintain shape stability during attachment to geometrics, thus offering consistent and precise feedback towards touching. Conversely, the FHP shows extraneous signals across states due to structural instability. Therefore, topological constraints ensure reliable signal acquisition in all interactive scenarios.

### Machine learning-assisted interaction recognition

**Figure 4.**
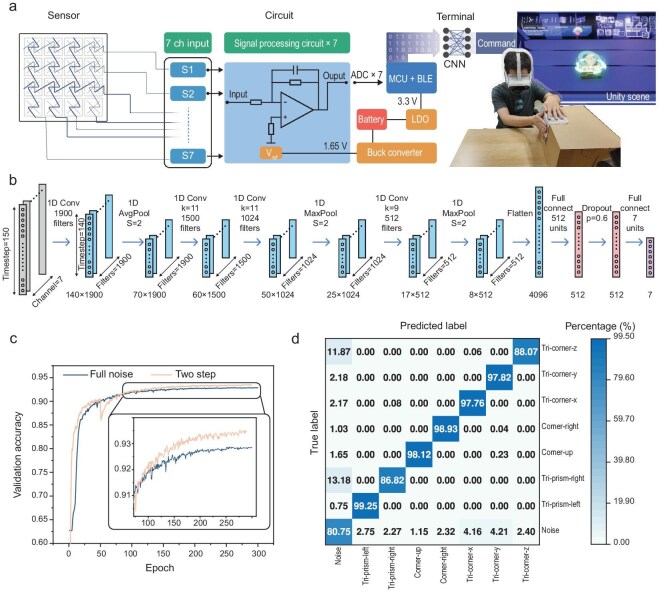
The composition of the 4 × 4 FTHP prototype system and the corresponding CNN model training process. (a) Prototype control flowchart. (b) Schematic diagram of the CNN structure corresponding to the transforming input mode. (c) Comparison of training speeds between the two-step training method and the one-step method with full noise. (d) Confusion matrix corresponding to the target actions of the folded input mode classification.

Machine learning was introduced to classify deformation and gesture recognition, mapping the signal outputs of the FTHP. As shown in Fig. 4a, the system architecture spans hardware to VR interaction. The hardware captures user actions, and after conditioning, the data are processed and collected by an analog-to-digital converter (ADC). The data are then relayed via Bluetooth to a CNN algorithm. The CNN’s inference results are integrated into a VR environment developed in Unity, enabling dynamic interactions based on the classification outcomes.

A custom PCB (20 × 30 × 4 mm^3^) handles signal processing and transmission. For gesture classification, CNNs target geometric shapes and interaction gestures common in VR (Table S1). We defined input rules for three states, assigning four gestures for the flat state, three geometric shapes for the folded state, and seven gestures for the transforming state. These input rules are generally sufficient to cover common interaction actions in a VR environment (Fig. S14). Considering the duration of each interaction state and the training accuracy, we customized CNN models for different states. For folded states involving longer interaction durations, a time window of 150 frames (approximately 3 s) was used as input data. Feature extraction was performed using a Conv1d, ReLU, Avg/Max Pool and fully connected architecture (Fig. 4b). In contrast, for flat and transforming states, we used input data from a 40-frame window and employed a Conv1d, ReLU, Max Pool and fully connected structure for training (Fig. S15). Each interaction action in all states was recorded for 500 epochs, and the resulting dataset was split into training, validation and test sets with a 6:2:2 ratio. A two-step training strategy was introduced to optimize training accuracy. First, the dataset was segmented and 80%–90% of the noise was removed. The training and testing sets were then divided into an 8:2 ratio and trained for 50 epochs to obtain the pre-trained parameters, based on which we continued training on the complete dataset for 250 additional iterations. This approach resulted in a significantly higher action recognition accuracy compared to directly training for 300 epochs (Fig. 4c). To reduce per-user data collection burden, we further evaluated subject-specific transfer learning from a cross-user base model, showing that high accuracy can be retained with substantially fewer samples per action (Fig. S18).

To justify the necessity of deep learning, we compared our CNN with traditional baselines (SVM and Random Forest) on a representative pose-mode dataset using the same split and windowing; both baselines show limited separability and substantially lower recall, consistent with overlapping feature distributions in principal component analysis (PCA) (Figs S19 and S20). After training the models for the three interaction states, the FTHP shows distinct classifications across 14 selected interactive actions (Fig. S16), with classification accuracies of 88.4%, 95.4% and 93.4% for the respective states (Fig. S17, Fig. 4d). The lower identification accuracy corresponding to the flat state compared with the other two is attributed to the slight amplitude of gestures. Tapping and sliding typically show similar changes in area and vertical compression stress, which leads to the presence of non-diagonal errors in the confusion matrix (Fig. S17). Overall, the high classification accuracies achieved across different states confirm the potential of the FTHP system to provide nuanced and reliable tactile feedback in VR applications.

### Verification of FTHP system in VR tasks

**Figure 5.**
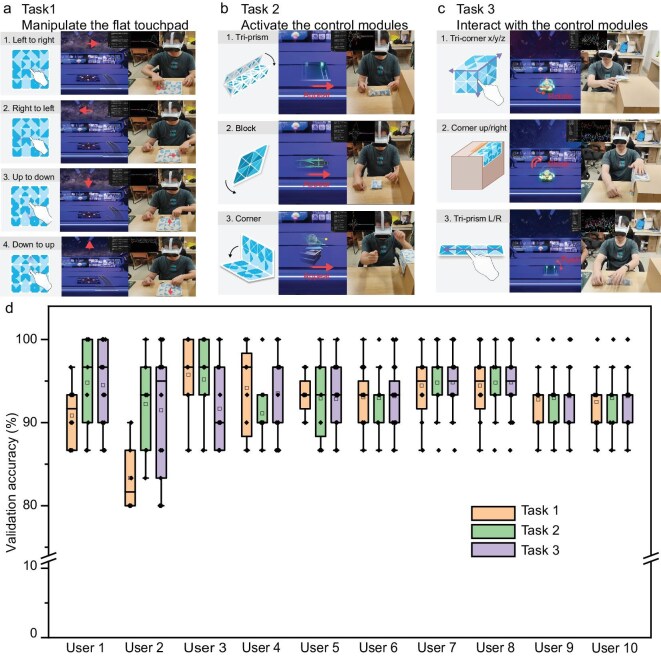
Recognition performance of the prototype in actual user scenarios. (a) Schematic of the task objective for users during the flat input test. (b) Schematic of the task objective for users during the transforming input test. (c) Schematic of the task objective for users during the folded input test. (d) The recognition accuracy for each task across different users.

A VR application focused on space exploration was developed to show the interactive capabilities of the FTHP. This application creates a virtual spaceship, where users can interact with objects using flat, folded, and transforming input gestures (Video S1). To account for individual differences in users’ handling habits, it is crucial to validate the robustness of the model across various interactions. We evaluated the performance of the FTHP, powered by a CNN, within the VR application by assessing the accuracy of different users in performing specific tasks across the three interaction modes. Three interactive tasks were designed, each corresponding to one of the three interaction modes, to validate the FTHP within specific VR interaction scenarios:

- Manipulate the flat touchpad: in the flat state, users control the view of a spacecraft using single-finger movements in four directions—up, down, left and right—adjusting the outlook from the control cabin window (Fig. 5a).
- Activate the control modules: the transforming state task involves users transforming the FTHP from a flat to various folded states—into a triangular prism, a small cube and a right-angled corner—to virtually activate the different tools (Fig. 5b).
- Interact with the control modules: for the folded state, users perform specific gestures on the ‘spaceship jump lever’ and a ‘planet model’ within the VR environment, including finger slides and taps to initiate a ‘spaceship space jump’ and rotate the model, respectively (Fig. 5c).

After a series of practice sessions (30 interactions per task), 10 users were evaluated on task accuracy using the FTHP. The results demonstrated high proficiency and robustness across multiple users. For the first task, the accuracies were 90.8%, 84.2%, 94.2%, 94.2%, 93.1%, 93.1%, 94.5%, 94.4%, 92.8% and 92.5%, respectively. For the second task, the accuracies were 94.8%, 92.2%, 95.2%, 91.1%, 92.9%, 92.9%, 94.8%, 94.8%, 92.9% and 92.9%, respectively. For the third task, the accuracies were 94.5%, 89.8%, 90.9%, 93.5%, 92.8%, 93.2%, 94.8%, 94.8%, 93.2% and 93.2% (Fig. 5d). These high task accuracies across all three interaction modes confirm the capability of FTHP for reliable multi-user interaction recognition.

## CONCLUSION

In conclusion, this work introduces the FTHP, establishing a new framework where engineered topology, rather than complex hardware, provides the core intelligence for interpreting physical interaction. This approach, realized through a synergy of origami-inspired structures and high-performance triboelectric yarns, transforms a simple textile into a programmable and high-fidelity VR interface. The design not only ensures the structural stability required for consistent tactile feedback but also encodes complex physical deformations into robust, classifiable electronic signatures. The high recognition accuracy achieved by our system validates this core principle, demonstrating a practical pathway to bypass the rigid one-to-one mapping of conventional haptic proxies. Looking forward, the principles of topological signal encoding could extend beyond VR to applications in soft robotics, smart wearables and augmented reality. Currently, due to limitations in sampling frequency and latency, FTHP may encounter issues when processing high-frequency interactions, such as simulating wing flapping. Due to its lack of autonomous deformation capability, our FTHP is not suitable as a solution for active haptic proxies. However, the system excels in scenarios utilizing metaphorical inputs based on deformation, where the act of folding itself triggers a virtual event. This research evolves the haptic proxy from a static physical mimic to a dynamic, information-rich interface, paving the way for more accessible, scalable and truly immersive virtual experiences.

## METHODS

The preparation and validation of this work are presented in the Supplementary data. These methods include the fabrication of triboelectric yarns, topological fabric prototype, the tests and characterization of our FTHP, circuit design and the validations in real-world applications.

### Ethical statement

Experiments regarding human research participants conducted in this article has gained approval from the Scientific and Technical Ethics Committee in Donghua University (SRSY202507010061).

## Supplementary Material

nwag041_Supplemental_Files
