# Supplementary material for: Fabric topological haptic proxy for interactive virtual reality
Source: Natl Sci Rev. 2026 Jan 23;13(5):nwag041. doi: 10.1093/nsr/nwag041 (PMC12916009; doi:10.1093/nsr/nwag041)
Supplement: nwag041_Supplemental_Files [file nwag041_supplemental_files.zip › Supplementary data.pdf]

Supplemental Information

Fabric Topological Haptic Proxy for Interactive Virtual Reality

Zhiyang Hu†, Tianzhan Liang†, Yuchen Wu†, Haoyu Wang†, Mingyu Zhou, Xinyan Lin, Leheng Chen, Shuqun An, Haojie Zhao,Yongqi Lou, Guoqing Zhang, Hongguo Gao, Fujie Li, Yuwen Zhu, Ling Zhang , Guanglin Zhang* , Liang-Wen Feng, Qi Wang*, Hengda Sun*, Xinge Yu, Hongzhi Wang, Jun Chen, Xiang-chen Li, Gang Wang*

Table of Contents

Supporting Information Text-- 4

Methods--- 7

Figure S1. Design draft of the FTHP prototype. -- 9

Figure S2. Design of one FTHP unit. ---10

Figure S3. The schematic of the process for fabricating multilayer triboelectric yarns.--------11

Figure S4. SEM morphology of the "S/Z" triboelectric yarn. --------------------------------------12

Figure S5. The working principle and simulation diagrams of triboelectric yarn.---------------13

Figure S6. The comparison of the output signals of 1L-Triboelectric yarn and 2L-Triboelectric yarn under the same test conditions.--14

Figure S7. The open-circuit voltage, short-circuit current, and output power magnitude of the 30 cm triboelectric yarn under various external loads. Each test point is taken from the average of five tests performed under the same conditions. ---------------------------------------15

Figure S8. The abrasion and washing tests of a triboelectric yarn. All the data set are based on the same yarn and fabric. --16

Figure S9. The signal strength variation of triboelectric yarns. ------------------------------------17

Figure S10. Response time of the triboelectric yarn.---18

Figure S11. Schematic representations of the impact of two distinct triboelectric yarn wiring methods on the recognition of actions.--19

Figure S12. Optical images of the FTHP featuring a detachable ribbon cable connection between the circuit board and the sensor yarn. ---20

Figure S13. Quantitative signal-to-noise ratio (SNR) and peak-amplitude analyses corresponding to the three representative interaction scenarios in Fig. 3C.-----------------------21

Figure S14. The schematic illustrations of the 14 actions recognized.-----------------------------22

Figure S15. 1D-CNN model structure for flat input and Folded input.----------------------------23

Figure S16. Classification clustering diagrams for all interaction mode actions.----------------24

Figure S17. Confusion matrices for different input modes.---25

Figure S18. Transfer learning results in the pose mode.---26

Figure S19. Performance of the SVM baseline on the pose classification task.------------------27

Figure S20. Performance of the Random Forest baseline on the pose classification task. -----28

Table S1. Statistical analysis of the geometric shapes and interactive gestures of haptic proxy interfaces commonly found in everyday VR usage environments. --------------------------------29

Movie S1 (separate file) --31

Supporting Information Text

Preparation of triboelectric yarn:

PVDF was dissolved in a mixed solvent of N, N-Dimethylformamide and acetone with a mass ratio of 6:4 to form an 18 wt% solution. The mixture was then stirred at 55°C for 10 hours to prepare the spinning solution. The core-sheath yarn was produced using a conjugate electrospinning machine (Yunfan Technology, Tianjin, China) with a voltage of ± 8 V, a flow rate of 0.8 ml h^{-1}, a winding speed of 200 rpm, and a collection speed of 2 rpm, using commercial silver-plated nylon yarn as the core. For multi-layer wrapped yarn, the spinning parameters for each layer remained unchanged, but the twisting direction of the nano yarn was reversed during spinning. The collected yarn was then dried in an oven at 60°C for 12 hours for further use.

Preparation of the topological fabric prototype:

The topological fabric block substrate was embroidered using a YueMei embroidery machine (WY1206C, YueMei), with commercially available nylon embroidery thread and PTFE sewing thread as materials. Embroidery was performed on commercially available plain weave cotton fabric following a preset pattern. The triboelectric yarn was sewn onto the topological fabric block substrate using a Brother Industrial Sewing Machine (NEXIO S-7220D, Brother), following a preset pattern.

Electrical output performance test

Unless specifically noted, electrical characterization was conducted using a 30 cm-long yarn wound around a pressure sensor with a 4cm diameter. An electrometer (6517B, Keithley) and a digital multimeter (DMM 6500, Keithley) were used in conjunction, with the 6517B collecting single-electrode TENG voltage, current, and charge parameters. These were then output to the DMM6500 through the 6517B's 2 V analog output channel and captured in the KickStart software. A linear motor impact tester (Naneng, Beijing) controlled the applied pressure (0-50 N) and collision frequency (1-3 Hz). For electrical characterization, the collision surfaces used were nitrile gloves unless otherwise specified. Materials specified for particular mention included cotton fabric, copper foil, polyethylene glycol terephthalate film, PTFE fabric, and nylon fabric used in electrical output tests with different frictional surfaces. Additionally, the electrical performance of the TENG yarns was investigated under varying humidity levels.

Mechanical performance test

The tensile strength of silver-coated nylon core yarn and TENG yarn with various winding numbers was evaluated using a filament yarn tensile tester (XL-1A, New Fiber Instrument, China). A pre-tension of 5 cN was applied, and each type of yarn underwent ten trials. The average value was taken as the corresponding strength.

Abrasion resistance test

A pilling tester (YG502, Laizhou Electron Instrument, China) was employed to assess the abrasion resistance of yarns embedded in fabric. The test was conducted in accordance with the GB/T 4802.2-1997 standard for pilling tests on textile fabrics using the Martindale method, applying a pressure of 590 cN and undergoing 150 pilling cycles and 150 fuzzing cycles. The fabric's condition post-abrasion test was observed and evaluated, followed by a subsequent evaluation of its electrical properties.

Circuit design and characteristics

The signal processing/transmission circuit board is primarily composed of a power chip (SPX3819), a battery charging chip (TP4057), an operational amplifier chip (RS8524), a microcontroller (STM32L433CCT6), and a Bluetooth module (RF-BM-BG22C3). The SPX3819 is a 500mA low-dropout regulator featuring 40 µV low noise and 1% high accuracy, used to convert battery voltage into 3.3 V to power the components. The TP4057 is a linear charger suitable for single-cell lithium batteries, with a maximum charging current of 600 mA, allowing the system's lithium battery to be charged via a USB-C interface on the circuit board without needing to remove the battery. The RS8524 is a quad-channel high-precision rail-to-rail operational amplifier. To achieve impedance transformation, we designed a signal conditioning circuit based on two RS8524 chips. One channel generates a bias voltage of 1.65 V, while the remaining seven channels, in conjunction with three resistors and one capacitor each, convert the triboelectric signal into a voltage signal that can be directly acquired by the ADC. Due to the single-point access circuit of the sensor, significant noise is introduced, which can be filtered out by the resistor and capacitor acting as a low-pass filter. The processed voltage signal is collected by the built-in 12bit ADC of the STM32L433CCT6. The STM32L433CCT6 is an ultra-low-power microcontroller based on the Arm® Cortex®-M4 with a floating-point unit, providing sufficient computational power for data acquisition and transmission. RF-BM-BG22C3 is an ultra-compact 8 × 8 mm Bluetooth 5.2

module with a receive sensitivity of -98.9 dBm under 1Mbit/s GFSK conditions and a TX current of 4.1 mA at 0 dBm output power. To power the circuit and maintain the system's compact size, we selected a small lithium battery (20 × 20 × 4 mm) to match our circuit board (20 × 30 × 4 mm), which has a capacity of 140 mAh, providing approximately 10 hours of operating time for the system.

ML model data collection and training

The VR task consists of three types of typical interactions, corresponding to the three states of Flat, Transforming and Folded. Users need to complete the task commands in different virtual scenes by sliding and folding. Each participant is required to perform about 30 training sessions to familiarize with the interface and operation mode before the formal test, and 500 sets of training samples are collected for each type of action for CNN model training, the specific data collection process is as follows:

1. Data acquisition: Ten users were selected for data acquisition after initial action training in an environment with a constant temperature of 20°C and a constant humidity of 45% RH to confirm that the type of signal output and signal quality were error-free. The sampling frequency was 50 Hz.

2. Touch pad mode (task 1): Users swiped on the fabric from top to bottom, bottom to top, left to right, and right to left, respectively, using their index and middle fingers, with the swipes covering four areas in the corresponding directions. Sliding was performed every 0.7 s and then again after the same time interval, with a total of 500 repetitions for each action.

3. Folded mode (task 2): During no movement folding, the user folded the fabric along the cross in the center, first upwards, then to the right again by pressing the center line, making sure that the fabric is flat after folding, the process should take 2.5 s. After 2 s, release the fabric to original shape. The releasing process should take 2.5 s. And the whole process was repeated after 2 s. The cross folding and return are divided into two movements, and each movement is repeated 500 epochs. During triangular prism folding, users held the fabric at the quarter furthest away from the transmission device and then folded it into a triangular prism. The process should take 2.5 s, and after 2 s it was released back to original shape, the process should take 1.5 s and was repeated after 2 s. The folding and returning of the triangular prism are divided into two movements, each of which was repeated 500 epochs. During lapping: Users pinched both sides of the fabric and rested it on the edge of a box with its corners on the centerline. The process should take 2.5 s, and after 2 s, users pinched both sides to flatten it on a table, the process should take 2

s and was repeated after 2 s. The resting and returning are divided into two movements, each of which was repeated 500 epochs.

After Folding (task 3): For forward and backward movement of the triangular prism, users grasped the folded triangular prism and moved it forward and backward with a movement time of 0.7 s and an interval of 0.7 s. The forward and backward movement was repeated 500 epochs. For sliding left and right movement of the triangular prism, users slided left and right on the folded triangular prism, the sliding time is 1 s, the interval is 1 s, and the left and right sliding are repeated 500 epochs.

All interaction data was collected at a fixed frequency of 50 Hz, and divided into training set, validation set and test set in the ratio of 6:2:2 (the window length of Flat/Transforming state is 40 frames, and the Folded state is 150 frames, the step size is 1). The sets were then input into the CNN model after a pre-processing process (normalization and filtering). The training strategy is divided into two stages: 50 rounds of pre-training with the noise-reduced subset was introduced to obtain the initial weights, and then 250 rounds of fine-tuning using the complete dataset with the loaded parameters was used to train three independent CNN models, ensuring the robustness and generalization ability of the training.

VR task validation

Each type of task was repeated 30 epochs, and the system automatically records the interaction process and analyzes the recognition. The specific validation process is as follows:

The model was integrated into the space exploration VR scene developed by Unity, and ten users were pre-trained to perform three types of tasks, which can manipulate the viewpoint of the spaceship to pan up, down, left, right and left in the state of the touchpad mode, activate/deactivate the corresponding tools in the folding/releasing mode, and operate the control module in folded state. Users repeated each action 30 epochs and the accuracy of the action was finally calculated.

Methods

Noise definition and reduction. In this work, "noise" refers to background windows that do not contain intentional interaction signals (i.e., no-action segments). Because real interactions occupy only a small portion of continuous recordings, background windows can dominate the dataset. To avoid bias toward the no-action class, we constructed a noise-reduced subset by randomly down-sampling background

windows until the background-to-total window ratio was below 20% (NOISE_RATIO = 0.2), which corresponds to removing approximately 80–90% of background samples.

Two-stage training strategy. We first pre-trained the 1D-CNN for 50 epochs using the noise-reduced subset to obtain a stable initialization. We then fine-tuned the network for 250 additional epochs on the complete dataset while loading the pre-trained weights. This two-stage strategy improves robustness to background noise while maintaining generalization across users.

Online sliding-window inference. Signals are sampled at 50 Hz (20 ms per frame). Online recognition uses a sliding window of 40 frames (~0.8 s) for Flat/Transforming and 150 frames (~3.0 s) for Folded. The window stride is 3 frames (~60 ms), yielding about 50/3 ≈ 16 predictions per second. Given that the lightweight 1D-CNN forward pass and Bluetooth/Unity processing are on the millisecond-to-tens-of-milliseconds scale, the end-to-end latency is dominated by the window duration (~0.8 s or ~3.0 s). Different window lengths are used because Folded-mode interactions typically span a longer fold–hold–unfold cycle, whereas Flat/Transforming gestures are faster and can be captured with shorter windows for better responsiveness.

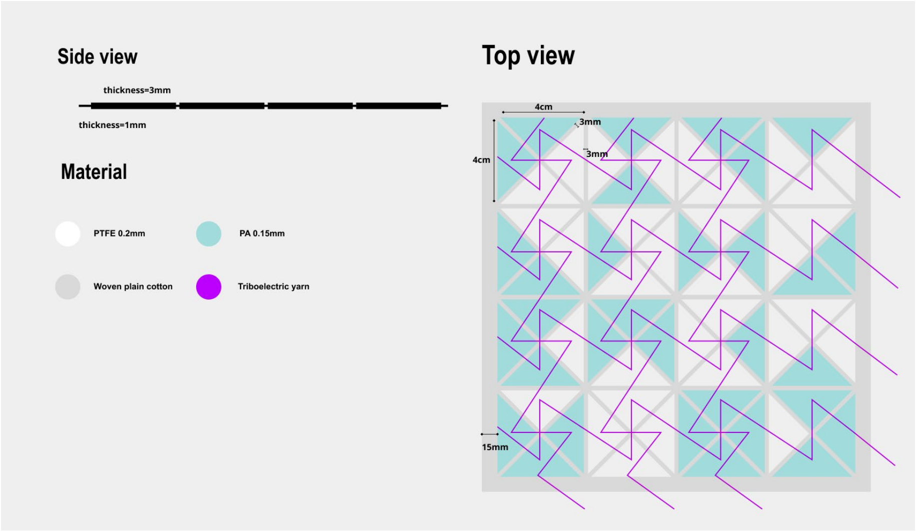

Figure S1. Design draft of the FTHP prototype.

Herein, the base is made of plain weave cotton fabric with a thickness of 1 mm. The rigid areas of the fabric segments are formed by embroidery patterns of PTFE (with a thread diameter of 0.2 mm) and nylon (with a thread diameter of 0.15 mm). Each rigid segment is an isosceles right triangle with a hypotenuse of 4 cm and a thickness of 3 mm. The conformable areas are 3 mm wide. A 15 mm cotton fabric margin is left around the edges for connecting the triboelectric yarn to the circuit board along the perimeter. The triboelectric yarn is sewn into each topological unit in a selected cross pattern, with the triboelectric yarns at the intersections being sewn as a single yarn in actual production. To verify the robustness of the wiring scheme, a vertical triboelectric yarn channel is intentionally omitted on the far-right side of the prototype to test whether a usable signal can be obtained when only one triboelectric yarn passes through four triangular blocks on a topological unit.

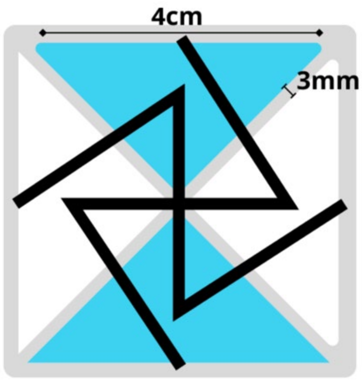

Figure S2. Design of one FTHP unit.

The transverse and longitudinal triboelectric yarns (black lines) traverse each segment of the topological unit, with blue and white regions representing substrates made of different materials. Notably, each unit features distinct substrate material combinations, enabling the specific identification of deformation behaviors occurring within that unit.

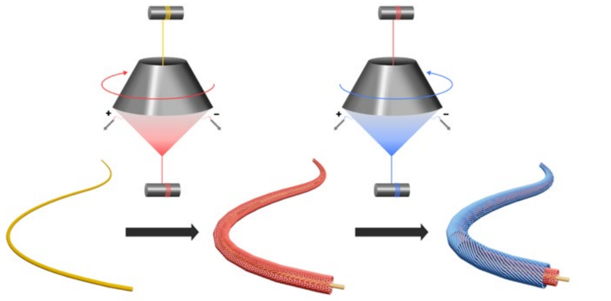

Figure S3. The schematic of the process for fabricating multilayer triboelectric yarns.

A 1L-Triboelectric yarn is formed by wrapping a layer of PVDF around the surface of silver-plated nylon yarn using conjugate electrospinning. To prepare a 2L-Triboelectric yarn, the 1L-Triboelectric yarn serves as the core yarn, around which a second layer of PVDF is formed using conjugate electrospinning, with the wrapping direction of the second PVDF layer being opposite to that of the first.

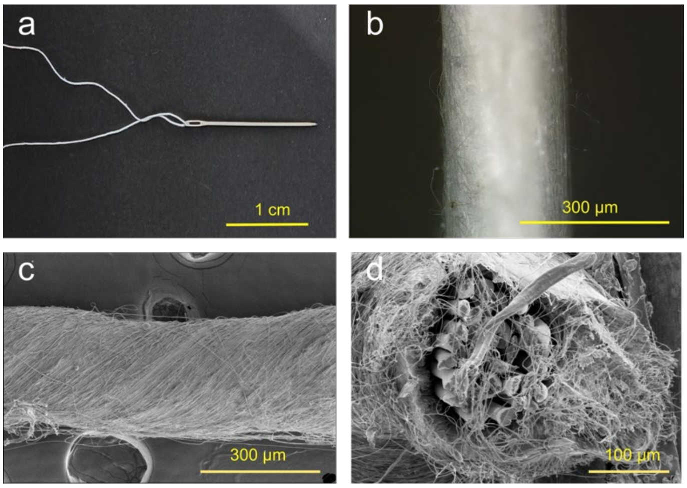

Figure S4. SEM morphology of the "S/Z" triboelectric yarn.

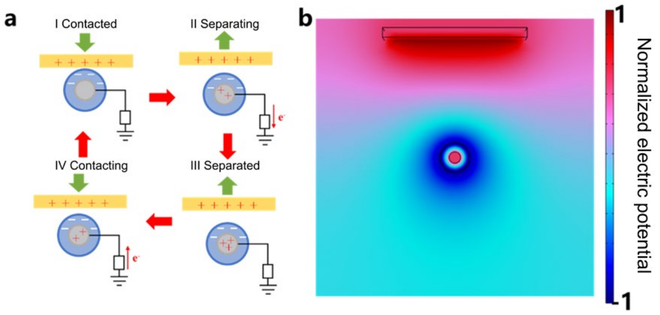

Figure S5. The working principle and simulation diagrams of triboelectric yarn.

(a) I-IV illustrate the single-electrode TENG working principle of triboelectric yarn. During the contact-separation process between the external friction surface and the surface of triboelectric yarn, the conductive core layer of triboelectric yarn generates induced charges that have the same polarity as the surface of the external contact. **(b)** A simulation diagram demonstrates the generation of induced charges within the core layer of triboelectric yarn. The electric potential in space is normalized within the range of [-1,1].

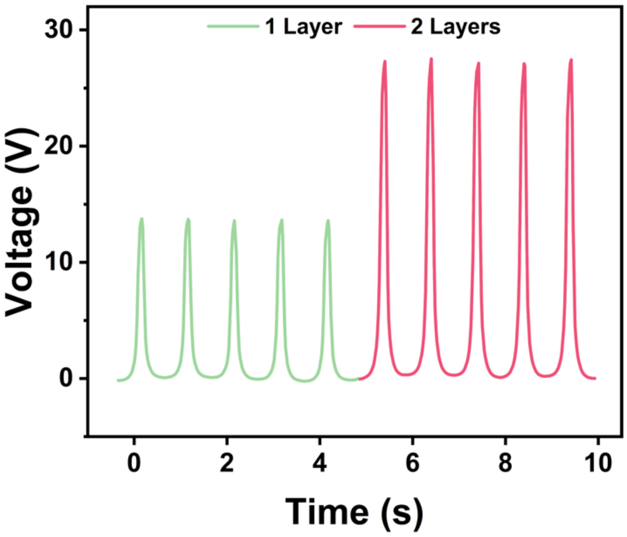

Figure S6. The comparison of the output signals of 1L-Triboelectric yarn and 2L-Triboelectric yarn under the same test conditions.

When a 30 cm triboelectric yarn was subjected to contact separation at a rate of 1 Hz with an external friction surface, the voltage outputs of 1L-Triboelectric yarn and 2L-Triboelectric yarn were approximately 14.3 V and 27.5 V, respectively.

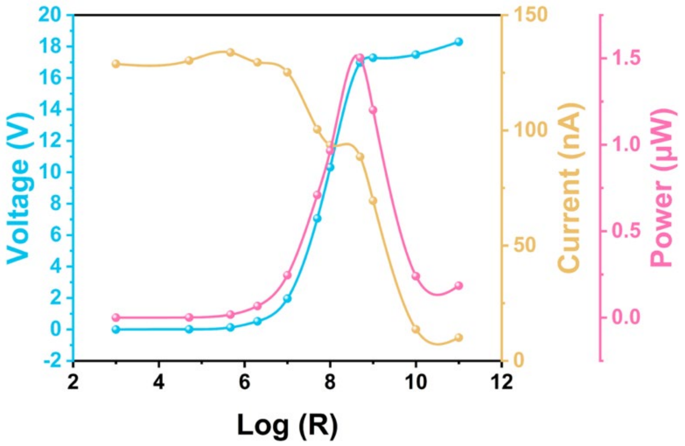

Figure S7. The open-circuit voltage, short-circuit current, and output power magnitude of the 30 cm triboelectric yarn under various external loads. Each test point is taken from the average of five tests performed under the same conditions.

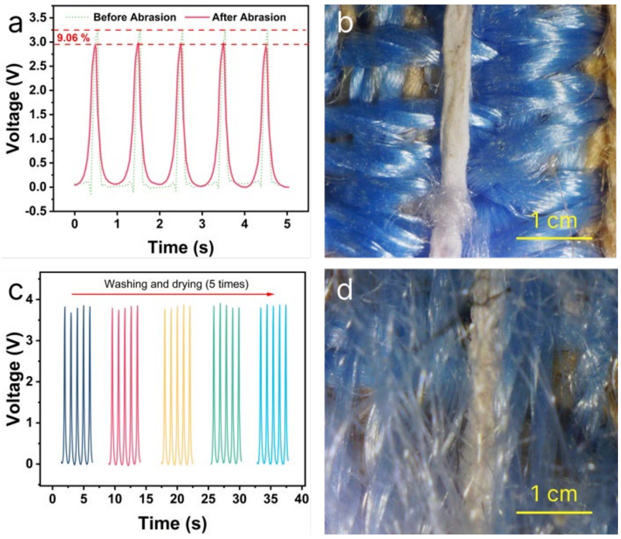

Figure S8. The abrasion and washing tests of a triboelectric yarn. All the data set are based on the same yarn and fabric.

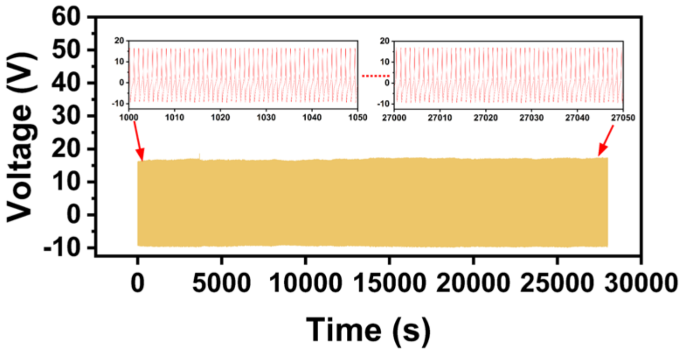

Figure S9. The signal strength variation of triboelectric yarns.

With the cyclic load exceeding a total of 27,000 cycles under a cyclic load pressure at 1 Hz

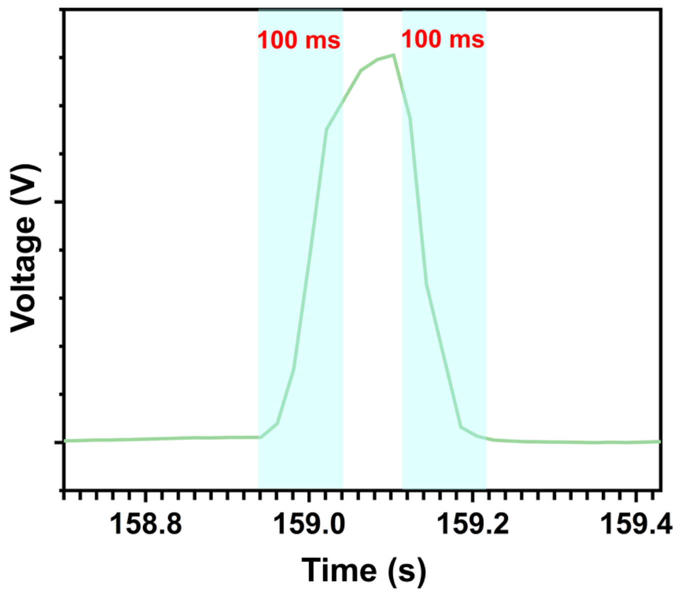

Figure S10. Response time of the triboelectric yarn.

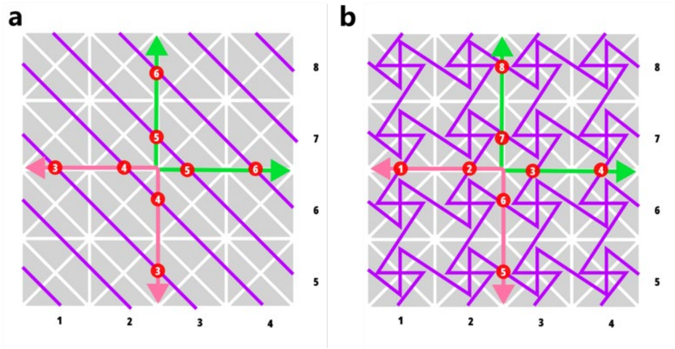

Figure S11. Schematic representations of the impact of two distinct triboelectric yarn wiring methods on the recognition of actions.

(a) a conventional wiring attempt, where actions directed along the red arrows traverse 3-4 channels, while those along the green arrows pass through 5-6 channels. **(b)** the wiring method designed based on topological units, where actions directed in the directions of the red and green arrows pass through different triboelectric yarn signal channels, thereby avoiding redundancy in the action signals.

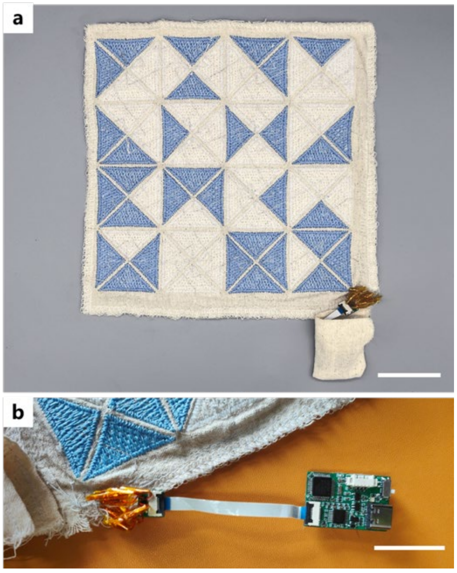

Figure S12. Optical images of the FTHP featuring a detachable ribbon cable connection between the circuit board and the sensor yarn.

(**a**) Optical image of the FTHP prototype. **(b)** The detachable ribbon cable connection, where the yarn ends are secured with copper tape and copper wire, with the copper wire soldered onto the terminals of the detachable ribbon cable interface. Scalebar: 2.5 cm.

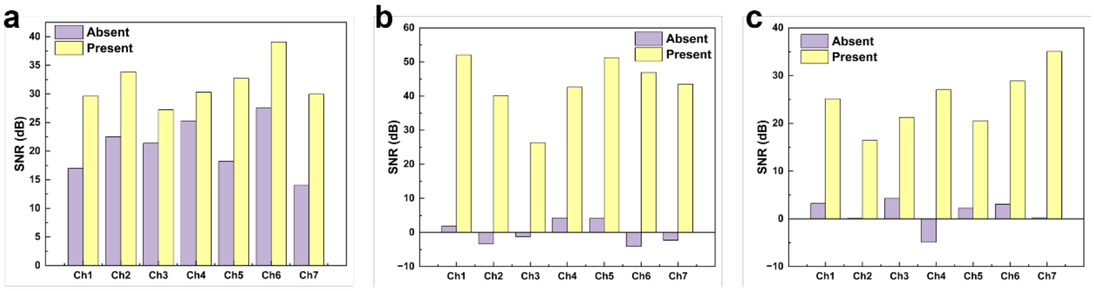

Figure S13. Quantitative signal-to-noise ratio (SNR) and peak-amplitude analyses corresponding to the three representative interaction scenarios in Fig. 3C.

(a) planar sliding, **(b)** fold-hold-release, and **(c)** sliding/self-deformation on a 3D topology.

For each condition (absent vs. present topological units), we first subtract the ~1.65 V DC offset, then define “noise” (unintended/background deformation) and “action” segments within a fixed time window. Channel-wise SNR is computed from the RMS amplitudes (SNR = 20 log10(RMS_action/RMS_noise)). The results show that adding topological units suppresses baseline fluctuations and self-deformation artifacts, leading to consistently higher SNR across channels even when some raw peaks become slightly lower, thereby increasing the contrast of intended inputs.

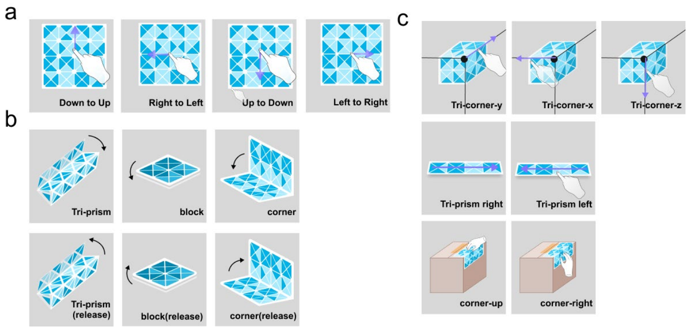

Figure S14. The schematic illustrations of the 14 actions recognized.

(a) Flat input actions, involving sliding movements in four directions (up, down, left, and right) on the surface of a flattened FTHP using the fingertip. **(b)** Transforming input actions, comprising the transformation from a flattened state to three geometric shapes, as well as the reversal back to the flattened state. **c,** Folded input actions, encompassing seven interactive gestures under three geometric shapes.

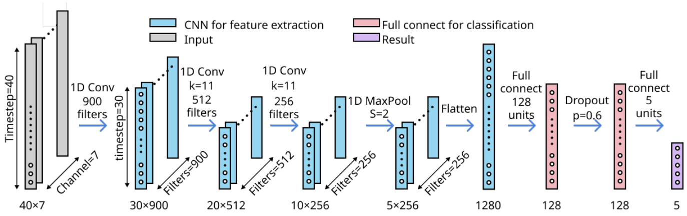

Figure S15. 1D-CNN model structure for flat input and Folded input.

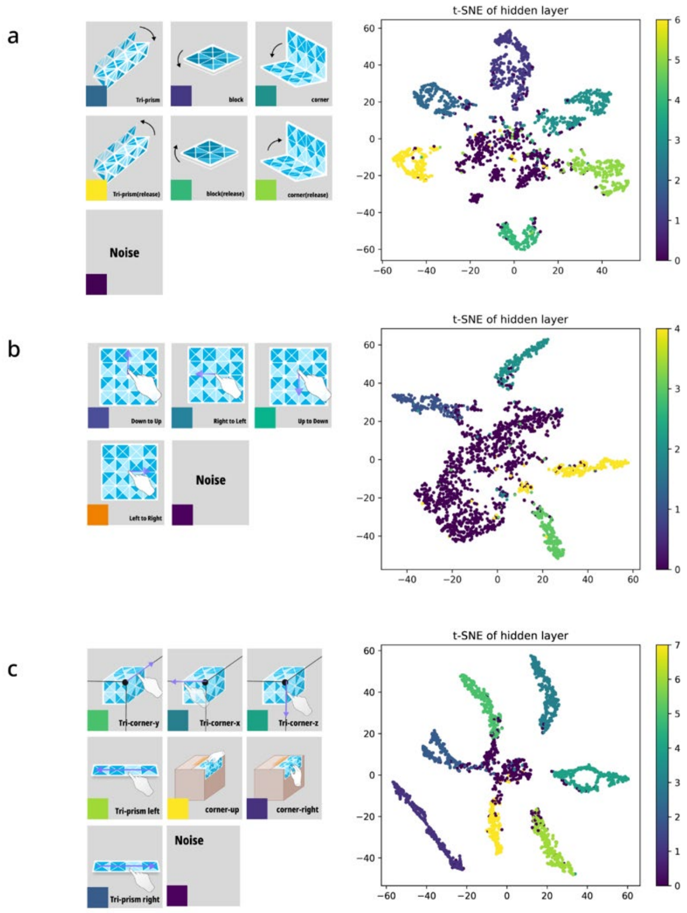

Figure S16. Classification clustering diagrams for all interaction mode actions.

(a) the interaction actions of the Transforming input mode and their corresponding clustering diagram. **(b)** the interaction actions of the Flat input mode and their corresponding clustering diagram. **(c)** the interaction actions of the Folded input mode and their corresponding clustering diagram.

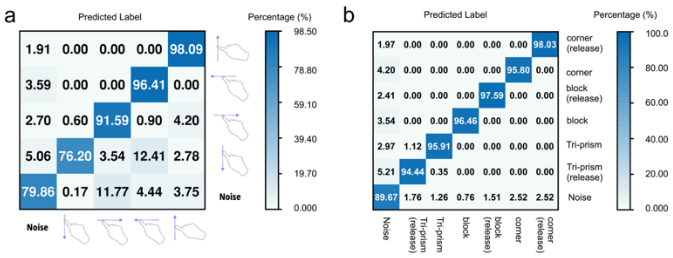

Figure S17. Confusion matrices for different input modes.

(a) the confusion matrix corresponding to flat input. **(b)** the confusion matrix corresponding to Folded input.

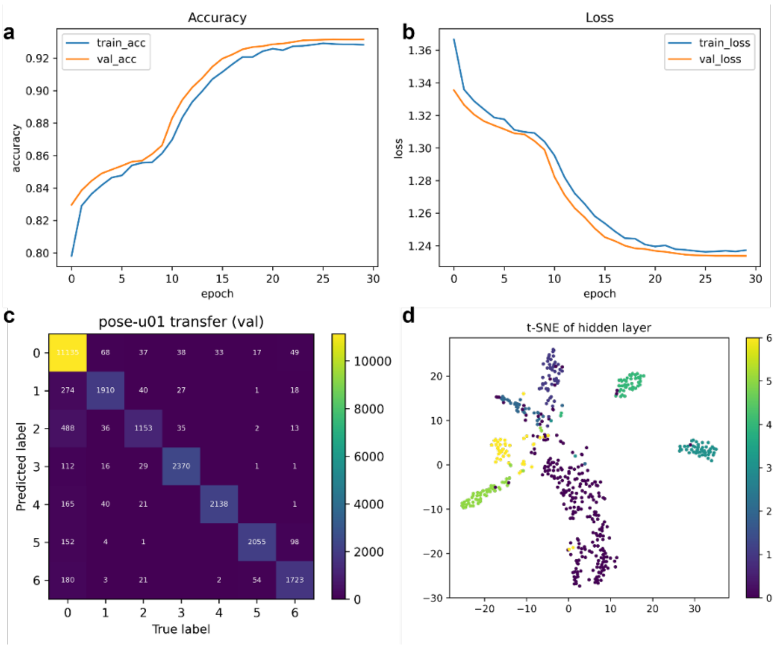

Figure S18. Transfer learning results in the pose mode.

(**a–b**) Training/validation curves during subject-specific fine-tuning with reduced samples per action. (**c**) Confusion matrix after transfer learning. (**d**) t-SNE visualization of latent features showing improved class separation after fine-tuning.

Transfer learning experiment in the pose mode. We trained a cross-user base CNN on the full pose dataset, then introduced a new subject with only 50 repetitions per action (vs. 500 in the full-data setting). During personalization, we froze the first two convolutional layers and fine-tuned the higher convolutional layers and fully connected layers (learning rate 1×10^{-4}, 30 epochs). (**a-b**) Training/validation curves show rapid convergence. (**c**) Confusion matrix after fine-tuning exhibits a strong diagonal with high per-class recall. (**d**) t-SNE visualization of latent features reveals well-separated clusters, indicating that the temporal features learned by the base model can be effectively adapted to a new user with ~1/10 of the data.

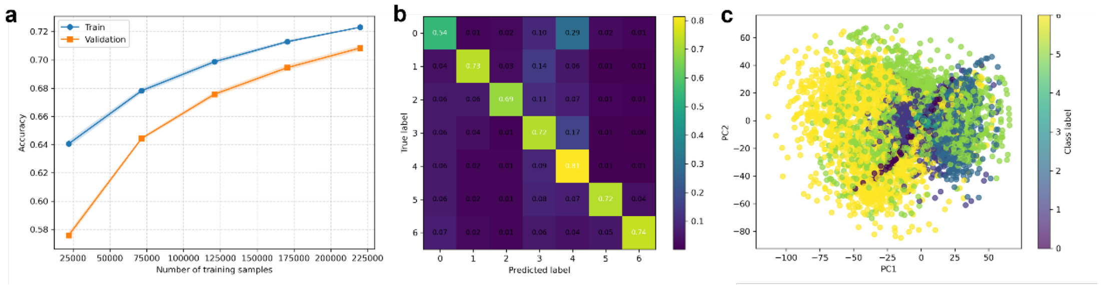

Figure S19. Performance of the SVM baseline on the pose classification task.

(**a**) Learning curves versus training samples. (**b**) Confusion matrix over the seven pose classes. (**c**) PCA projection of flattened input features colored by pose label.

SVM baseline in the pose mode using the same sliding-window segmentation as the CNN. Each window' s 7-channel time series is flattened and standardized, and a multi-class SVM with an RBF kernel is trained. (**a**) Learning curves show minimal overfitting, suggesting performance is limited by model capacity and the flattened feature representation. (**b**) The confusion matrix shows that many pose classes remain partially confused, yielding an average recall of ~70% across the seven classes. (**c**) PCA projection of the flattened features shows substantial overlap among classes, consistent with the limited separability available to shallow classifiers.

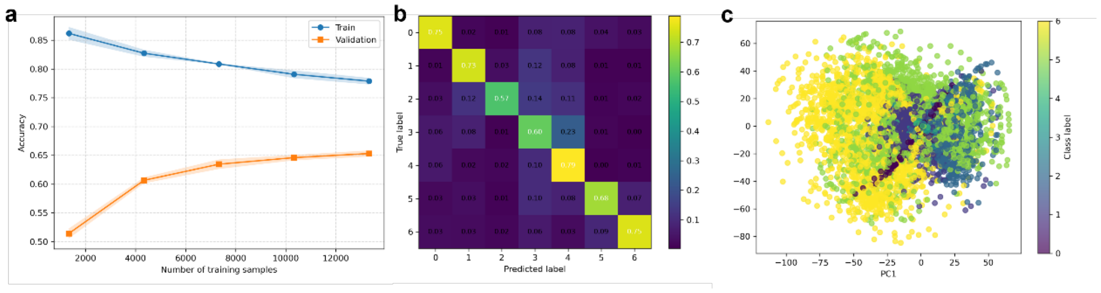

Figure S20. Performance of the Random Forest baseline on the pose classification task.

(a) Learning curves versus training samples. **(b)** Confusion matrix over the seven pose classes. **(c)** PCA projection of flattened input features colored by pose label.

Random Forest (RF) baseline in the pose mode using the same flattened and standardized sliding-window features. (**a**) Learning curves show only mild overfitting, indicating that generalization is mainly limited by feature representation. (**b**) The confusion matrix similarly indicates partial confusions between several pose classes and an average recall of ~70%. (**c**) PCA projection shows overlapping class distributions, supporting that low-level flattened features provide insufficiently separable structure for RF, in contrast to the CNN's ability to learn discriminative temporal patterns.

Table S1. Statistical analysis of the geometric shapes and interactive gestures of haptic proxy interfaces commonly found in everyday VR usage environments.

Typical Interaction Item	Typical Interaction Interface	Function	Type
Bed	Bed surface	Sit on flat surface	Flexible surface
	Bedside	Kick/slide with feet	Hard surface
Sofa	Cushion	Lean against	Soft cuboid
	Seat cushion	Sit on flat surface	Soft cuboid
	Armrest	Place arm/rest	Flexible surface
Table	Tabletop	Rest arms	Hard surface
	Table leg	Kick/slide with feet	Hard cylindrical
Stool/Chair	Seat cushion	Sit on flat surface	Hard surface
	Chair leg	Kick/slide with feet	Hard cylindrical
	Armrest	Place arm/rest	Hard surface
Pillow	Surface	Rub/stretch/pinch/squeeze	Soft cuboid
Container	Inside	Scoop/tilt/place	Hard surface
	Outside	Hard surface	Hard surface
Carpet	Surface	Step on/slide with feet	Hard surface
Lighting fixtures	Lampshade	Rotate cylindrical	Hard surface
	Lamp cord	Pull/tug/swing	Cord-like material

Cabinet	Cuboid	Place/take out items	Hard cuboid
	Door	Rotate along axis	Hard surface
Decorative painting	Vertical surface	Slide on surface	Hard surface
Wall	Vertical surface	Slide on surface	Hard surface
Door	Vertical surface	Rotate along axis	Hard surface
Television	TV frame/body	Power on/off	Hard frame and corners
	Vertical surface	Slide on surface	Hard surface
Bookshelf	Cuboid	Place/take out items	Hard surface
Computer	Monitor vertical surface	Slide on surface	Hard surface
	Computer frame/body	Power on/off	Hard frame and corners
Speaker	Cuboid	Sense vibrations	Hard surface
Electric fan	Cylinder	Rotate direction	Hard curved surface
	Control panel	Turn knob	Irregular surface
Musical instrument	Musical instrument	Play/tune	Irregular surface
Electric kettle	Pillar	Hold handle/tilt	Hard curved surface

Movie S1 (separate file)

Virtual objects interact with users by employing the FTHP through Flat input, Folded input, and Transforming input gestures.
